# Supplementary material for: Molecular Polariton Dynamics in Realistic Cavities
Source: J Chem Theory Comput. 2025 Oct 3;21(19):9823–31. doi: 10.1021/acs.jctc.5c01318 (PMC12529916; doi:10.1021/acs.jctc.5c01318)
Supplement: Supplementary file 1 [file ct5c01318_si_001.pdf]

Supporting Information: Molecular polariton dynamics in realistic cavities

Carlos M. Bustamante,[*,†] Franco P. Bonafé,[†] Maxim Sukharev,[‡,¶] Michael Ruggenthaler,[†] Abraham Nitzan,[§] and Angel Rubio[†]

†*Max Planck Institute for the Structure and Dynamics of Matter and Center for Free-Electron Laser Science, Luruper Chaussee 149, Hamburg 22761, Germany*

‡*College of Integrative Sciences and Arts, Arizona State University, Mesa, Arizona 85212, United States*

¶*Department of Physics, Arizona State University, Tempe, Arizona 85287, United States*

§*Department of Chemistry, University of Pennsylvania, Philadelphia, Pennsylvania 19104, United States*

E-mail: carlos.bustamante@mpsd.mpg.de

S1 Semi-classical limit of the Pauli-Fierz Hamiltonian and quantum-electrodynamical density-functional theory

We start with the Pauli-Fierz Hamiltonian in SI units (we follow in this SI Ref. [1]) given as

$$
\begin{aligned}
\hat{H}_{\mathrm{PF}} = & \underbrace{\sum_{l=1}^{N_e} \frac{1}{2m}\left(-\mathrm{i}\hbar\nabla_{\mathbf{r}_l} + \frac{|e|}{c}\hat{\mathbf{A}}_{\perp}(\mathbf{r}_l)\right)^2 + \frac{|e|\hbar}{2m}\boldsymbol{\sigma}_l\cdot\hat{\mathbf{B}}(\mathbf{r}_l) + \frac{1}{2}\sum_{l=1}^{N_e}\sum_{m\neq l}^{N_e}\frac{e^2}{4\pi\epsilon_0|\mathbf{r}_l-\mathbf{r}_m|}}_{\text{electronic sector + photon}-\text{electron interaction}} \\
& \underbrace{+\sum_{l=1}^{N_n}\frac{1}{2M_l}\left(-\mathrm{i}\hbar\nabla_{\mathbf{R}_l} - \frac{Z_l|e|}{c}\hat{\mathbf{A}}_{\perp}(\mathbf{R}_l)\right)^2 - \frac{Z_l|e|\hbar}{2M_l}\boldsymbol{S}_l\cdot\hat{\mathbf{B}}(\mathbf{R}_l) + \frac{1}{2}\sum_{l=1}^{N_n}\sum_{m\neq l}^{N_n}\frac{Z_l Z_m e^2}{4\pi\epsilon_0|\mathbf{R}_l-\mathbf{R}_m|}}_{\text{nuclear/ionic sector + photon}-\text{nucleus/ion interaction}} \qquad (1) \\
& \underbrace{-\sum_{l=1}^{N_e}\sum_{m=1}^{N_n}\frac{Z_m e^2}{4\pi\epsilon_0|\mathbf{r}_l-\mathbf{R}_m|}}_{\text{electron}-\text{nucleus/ion interaction}} \underbrace{+\sum_{\lambda=1}^{2}\int \hbar\omega_{\mathbf{k}}\hat{a}^{\dagger}(\mathbf{k},\lambda)\hat{a}(\mathbf{k},\lambda)\mathrm{d}\mathbf{k}}_{\text{free photon field energy}}.
\end{aligned}
$$

Here, the first line describes the electronic sector of the theory and its interaction induced by the Coulomb-gauged photon field with the quantized vector potential (in units of Volts)

$$
\hat{\mathbf{A}}_{\perp}(\mathbf{r}) = \sqrt{\frac{\hbar c^2}{\epsilon_0(2\pi)^3}}\sum_{\lambda=1}^{2}\int_{\mathbb{R}^3}\frac{\boldsymbol{\epsilon}(\mathbf{k},\lambda)}{\sqrt{2\omega_{\mathbf{k}}}}\left(\hat{a}(\mathbf{k},\lambda)\exp(\mathrm{i}\mathbf{k}\cdot\mathbf{r}) + \hat{a}^{\dagger}(\mathbf{k},\lambda)\exp(-\mathrm{i}\mathbf{k}\cdot\mathbf{r})\right)\mathrm{d}\mathbf{k}, \qquad (2)
$$

where $\boldsymbol{\epsilon}(\mathbf{k},\lambda)$ are the two transverse polarization vectors, and $\hat{a}(\mathbf{k},\lambda)$ and $\hat{a}^{\dagger}(\mathbf{k},\lambda)$ the usual bosonic annihilation and creation field operators. The magnetic field operator is defined by

$$
\hat{\mathbf{B}}(\mathbf{r}) = \tfrac{1}{c}\nabla\times\hat{\mathbf{A}}_{\perp}(\mathbf{r}) \qquad (3)
$$

and $\boldsymbol{\sigma}$ is a vector of spin-1/2 Pauli matrices. The second line describes the nuclei (or more generally ions) as effective quantum particles with an effective mass M_l, an effective charge $Z_l|e|$ and an effective spin S, which gives rise to a vector of spin matrices $\boldsymbol{S}_l$. The last line

describes the longitudinal interaction between the nuclei/ions and the electrons, and finally the energy of the free electromagnetic field. We note that all the masses above are *bare* masses that depend on the ultra-violet regularization of the interaction.

The mean-field (quantum-mechanical) Maxwell-Pauli theory is then found in two consecutive approximation steps. First, we rewrite the coupling terms as expectation value $\mathbf{A}_{\perp}(\mathbf{r}, t)$ and fluctuations $\Delta\hat{\mathbf{A}}_{\perp}(\mathbf{r}, t)$ by

$$\hat{\mathbf{A}}_{\perp}(\mathbf{r}) = \underbrace{\langle\Psi(t)|\hat{\mathbf{A}}_{\perp}(\mathbf{r})|\Psi(t)\rangle}_{=\mathbf{A}_{\perp}(\mathbf{r},t)} + \underbrace{\hat{\mathbf{A}}_{\perp}(\mathbf{r}) - \mathbf{A}_{\perp}(\mathbf{r}, t)}_{=\Delta\hat{\mathbf{A}}_{\perp}(\mathbf{r},t)} \tag{4}$$

for the fully coupled light-matter wave function $|\Psi(t)\rangle$, and accordingly for the magnetic field. We then assume that the fluctuations affect the matter subsystem in the same way as the unperturbed vacuum fluctuations, such that we can approximately subsume them in the usual *observable* masses of the particles. That is, we replace

$$m \rightarrow m_e \quad \text{and} \quad M_l \rightarrow M_{n,l} \tag{5}$$

in the matter Hamiltonian. After the mass renormalization, only the expectation value $\mathbf{A}_{\perp}(\mathbf{r}, t)$ is left to couple to the particles. This implies that after mass-renormalization we make a tensor-product ansatz $|\Psi(t)\rangle \approx |\psi_{\mathrm{M}}(t)\rangle \otimes |\phi_{\mathrm{ph}}(t)\rangle$, with $|\psi_{\mathrm{M}}(t)\rangle$ a pure matter wavefunction and $|\phi_{\mathrm{ph}}(t)\rangle$ a pure photon wavefunction. If we are not interested in the photon statistics, we can replace the mean-field coupled photon Hamiltonian by just the inhomogeneous Maxwell equation in Coulomb gauge without any further approximation.

Thus we have to solve a time-dependent Schrödinger equation with the Pauli Hamiltonian

$$\hat{H}_{\rm P}(t) = \sum_{l=1}^{N_e} \frac{1}{2m_e}\left(-\mathrm{i}\hbar\nabla_{\mathbf{r}_l} + \frac{|e|}{c}\mathbf{A}_{\perp}(\mathbf{r}_l,t)\right)^2 + \frac{|e|\hbar}{2m}\boldsymbol{\sigma}_l\cdot\mathbf{B}(\mathbf{r}_l,t) + \frac{1}{2}\sum_{l=1}^{N_e}\sum_{m\neq l}^{N_e}\frac{e^2}{4\pi\epsilon_0|\mathbf{r}_l-\mathbf{r}_m|}$$
$$+\sum_{l=1}^{N_n}\frac{1}{2M_{n,l}}\left(-\mathrm{i}\hbar\nabla_{\mathbf{R}_l} - \frac{Z_l|e|}{c}\mathbf{A}_{\perp}(\mathbf{R}_l,t)\right)^2 - \frac{Z_l|e|\hbar}{2M_l}\boldsymbol{S}_l\cdot\mathbf{B}(\mathbf{R}_l,t) + \frac{1}{2}\sum_{l=1}^{N_n}\sum_{m\neq l}^{N_n}\frac{Z_l Z_m e^2}{4\pi\epsilon_0|\mathbf{R}_l-\mathbf{R}_m|}$$
$$-\sum_{l=1}^{N_e}\sum_{m=1}^{N_n}\frac{Z_m e^2}{4\pi\epsilon_0|\mathbf{r}_l-\mathbf{R}_m|}, \tag{6}$$

self-consistently with the inhomogeneous Maxwell equation

$$\left(\tfrac{1}{c^2}\partial_t^2 - \nabla^2\right)\mathbf{A}_{\perp}(\mathbf{r},t) = \mu_0 c\mathbf{J}_{\perp}(\mathbf{r},t), \tag{7}$$

where $\mathbf{J}_{\perp}(\mathbf{r},t)$ is the transverse part of the current expectation value of the above Pauli Hamiltonian. The latter equation can be rewritten in the macroscopic form given in Eq. (1) in the main text.

Now, for both, the full Pauli-Fierz Hamiltonian as well as the Pauli Hamiltonian we can define corresponding time-dependent density-functional theories. In both cases the basic mappings and approximations are found from two coupled equations of motion. In the case of the Pauli-Fierz Hamiltonian we consider the coupled pair

$$\partial_t\mathbf{J}_{\rm PF}(\mathbf{r},t) = \frac{\mathrm{i}}{\hbar}\langle\Psi(t)|[\hat{H}_{\rm PF},\hat{\mathbf{J}}_{\rm PF}(\mathbf{r})]|\Psi(t)\rangle \quad \text{and} \quad \left(\tfrac{1}{c^2}\partial_t^2 - \nabla^2\right)\mathbf{A}_{\perp}(\mathbf{r},t) = \mu_0 c\mathbf{J}_{\rm PF,\perp}(\mathbf{r},t) \tag{8}$$

and in the case of the Maxwell-Pauli theory

$$\partial_t\mathbf{J}(\mathbf{r},t) = \frac{\mathrm{i}}{\hbar}\langle\psi_{\rm M}(t)|[\hat{H}_{\rm P}(t),\hat{\mathbf{J}}(\mathbf{r},t)]|\psi_{\rm M}(t)\rangle \quad \text{and} \quad \left(\tfrac{1}{c^2}\partial_t^2 - \nabla^2\right)\mathbf{A}_{\perp}(\mathbf{r},t) = \mu_0 c\mathbf{J}_{\perp}(\mathbf{r},t). \tag{9}$$

Since $\mathbf{J}_{\rm PF}(\mathbf{r},t) \to \mathbf{J}(\mathbf{r},t)$ upon mass-renormalization and mean-field coupling, as discussed above, quantum-electrodynamical density-functional theory (the density-functional reformu-

lation of the Pauli-Fierz theory) reduces to a density-functional reformulation of the coupled Maxwell-Pauli theory and subsequent approximations such as coupled Maxwell-Schrödinger theory,[2,3] which is the fundamental theory on which the scheme in the main text is based on.

S2 Electric field visualization in 2D-simulations

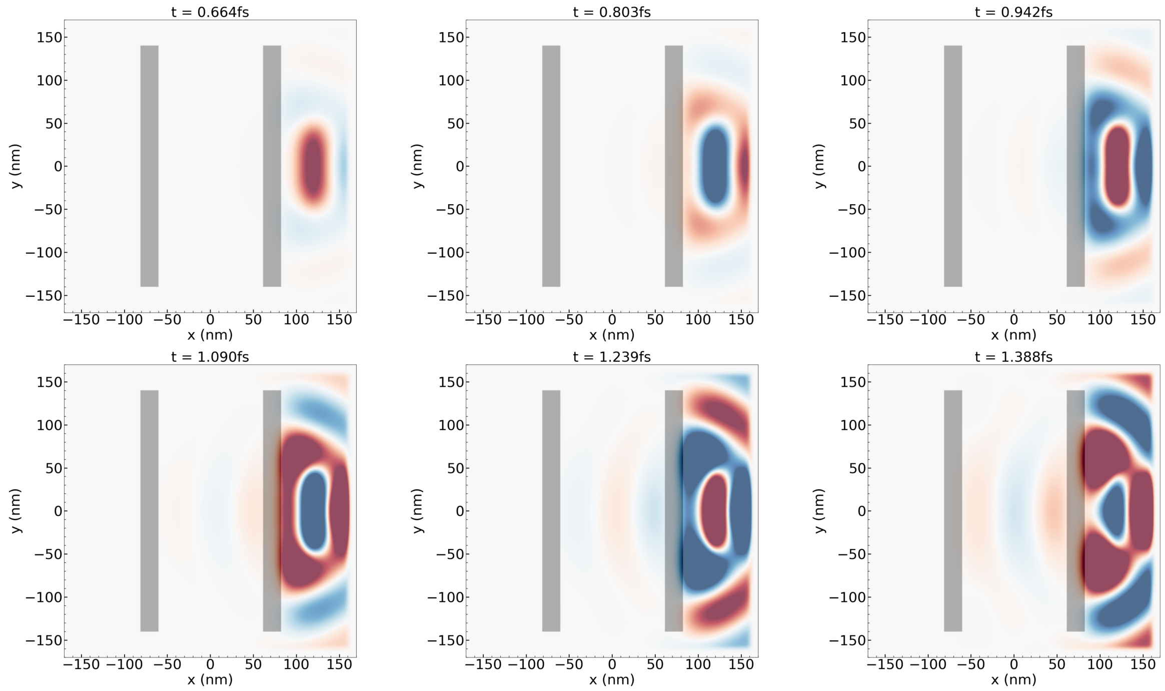

Figure S1: Snapshots of the z-component of the electric field during the first instants of the simulation. The field is created by the external source shaped by 9 point-like Gaussian pulses symmetrically placed on a line at x = 120 nm, symmetrically separated by 10 nm, with respect to $y = 0$. The pulses have a frequency of 14 eV and a FWHM of 0.588 fs.

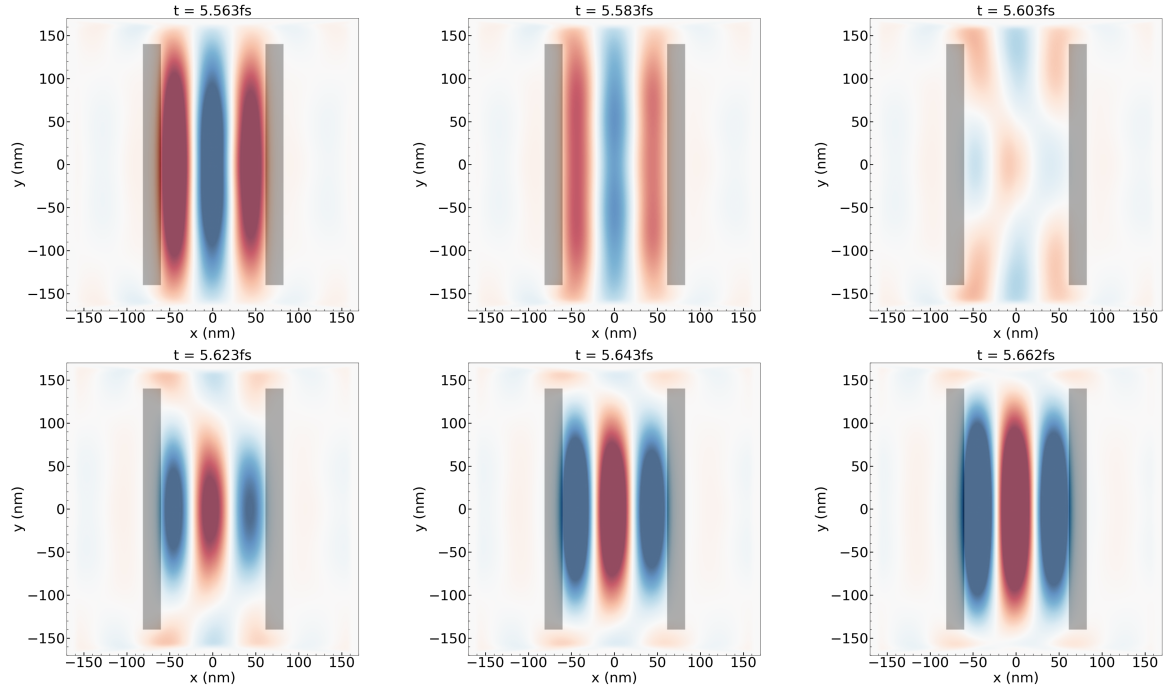

Figure S2: Snapshots of the z-component of the electric field after the excitation has finished. At this stage, the shape of the third cavity mode, the one that interacts strongly with the N_2 electronic excitation, can be observed.

References

(1) Ruggenthaler, M.; Sidler, D.; Rubio, A. Understanding polaritonic chemistry from ab initio quantum electrodynamics. *Chemical Reviews* **2023**, *123*, 11191–11229.

(2) Ruggenthaler, M.; Flick, J.; Pellegrini, C.; Appel, H.; Tokatly, I. V.; Rubio, A. Quantum-electrodynamical density-functional theory: Bridging quantum optics and electronic-structure theory. *Physical Review A* **2014**, *90*, 012508.

(3) Jestädt, R.; Ruggenthaler, M.; Oliveira, M. J.; Rubio, A.; Appel, H. Light-matter interactions within the Ehrenfest–Maxwell–Pauli–Kohn–Sham framework: fundamentals, implementation, and nano-optical applications. *Advances in Physics* **2019**, *68*, 225–333.
